# Evidence-based planning and costing palliative care services for children: novel multi-method epidemiological and economic exemplar

**DOI:** 10.1186/1472-684X-12-18

**Published:** 2013-04-25

**Authors:** Jane Noyes, Rhiannon Tudor Edwards, Richard P Hastings, Richard Hain, Vasiliki Totsika, Virginia Bennett, Lucie Hobson, Gareth R Davies, Ciarán Humphreys, Mary Devins, Llinos Haf Spencer, Mary Lewis

**Affiliations:** 1 School of Healthcare Sciences, Bangor University, Bangor, UK; 2 Institute for Medical and Social Care Research, Bangor University, Bangor, UK; 3 School of Psychology, Bangor University, Bangor, UK; 4 School of Medical Sciences, Bangor University, Bangor, UK; 5 National Institute for Social Care and Health Research Clinical Research Collaboration, North Wales Research Network, North Wales, UK; 6 Public Health Intelligence Analyst, Public Health Wales NHS Trust, Wales, UK; 7 Public Health Wales NHS Trust, Wales, UK; 8 Paediatric Palliative Medicine, Our Lady’s Children’s Hospital Crumlin and The Coombe Women & Infants University Hospital, Dublin, Eire, Ireland; 9 Royal United Hospital Bath NHS Trust, Bath, UK

**Keywords:** Children, Palliative care, Life-limiting illness, Evidence-based, Commissioning framework, Health economics, Cost, Health services research

## Abstract

**Background:**

Children’s palliative care is a relatively new clinical specialty. Its nature is multi-dimensional and its delivery necessarily multi-professional. Numerous diverse public and not-for-profit organisations typically provide services and support. Because services are not centrally coordinated, they are provided in a manner that is inconsistent and incoherent. Since the first children’s hospice opened in 1982, the epidemiology of life-limiting conditions has changed with more children living longer, and many requiring transfer to adult services. Very little is known about the number of children living within any given geographical locality, costs of care, or experiences of children with ongoing palliative care needs and their families. We integrated evidence, and undertook and used novel methodological epidemiological work to develop the first evidence-based and costed commissioning exemplar.

**Methods:**

Multi-method epidemiological and economic exemplar from a health and not-for-profit organisation perspective, to estimate numbers of children under 19 years with life-limiting conditions, cost current services, determine child/parent care preferences, and cost choice of end-of-life care at home.

**Results:**

The exemplar locality (North Wales) had important gaps in service provision and the clinical network. The estimated annual total cost of current children’s palliative care was about £5.5 million; average annual care cost per child was £22,771 using 2007 prevalence estimates and £2,437- £11,045 using new 2012/13 population-based prevalence estimates. Using population-based prevalence, we estimate 2271 children with a life-limiting condition in the general exemplar population and around 501 children per year with ongoing palliative care needs in contact with hospital services. Around 24 children with a wide range of life-limiting conditions require end-of-life care per year. Choice of end-of-life care at home was requested, which is not currently universally available. We estimated a minimum (based on 1 week of end-of-life care) additional cost of £336,000 per year to provide end-of-life support at home. Were end-of-life care to span 4 weeks, the total annual additional costs increases to £536,500 (2010/11 prices).

**Conclusions:**

Findings make a significant contribution to population-based needs assessment and commissioning methodology in children’s palliative care. Further work is needed to determine with greater precision which children in the total population require access to services and when. Half of children who died 2002-7 did not have conditions that met the globally used children's palliative care condition categories, which need revision in light of findings.

## Background

Families who have children with life-limiting conditions and complex healthcare needs require early and ongoing support from diagnosis onwards with their child’s health and social care. Ongoing and timely support is also designed to minimise the wider impacts on the family [1]. In a children’s context, this type of support is called ‘palliative care’. As there is often uncertainty about a child’s illness trajectory, children’s palliative care may combine palliative care with cure oriented treatment.

Due to advances in medical and nursing care, more children with complex healthcare needs live longer and require more palliative care services for longer periods of time [2,3]. Some children need access to palliative care services and support over decades and into adulthood [3,4].

In response to a rapidly growing population of children with life-limiting conditions, the United Kingdom (UK) has led the way in developing children’s palliative care as an internationally recognised specialty [5]. Helen House opened in Oxford, UK, in 1982 as the world's first children's hospice and many others have now opened globally based on a similar model [6]. Development and expansion of UK-based children’s general, specialist and outreach community nursing services providing palliative care followed, with pioneering services such as the Lifetime Service in Bath developing a new model of family-based care and psychological support that has been replicated elsewhere [7-10]. There is now a small group of senior doctors who provide strong leadership in children’s palliative care medicine (including Hain co-author), and who have organised the specialty into managed palliative care networks [5,11,12].

An independent review of palliative care services for children and young people in England commissioned by the Department of Health was conducted in 2006–2007 [13]. Similar reviews were conducted in Scotland and Wales [14,15]. These reviews consistently concluded that services had developed in a sporadic and unplanned way, with funding for this patient group often short-term. Clinical service frameworks and guidance [16] now contain clear objectives to develop costed children’s palliative care services on the basis of incidence and prevalence in each locality, and child/family preferences. Achieving these objectives is not however possible without integrating existing evidence and methodological development and further research described in this epidemiological and economic exemplar. Nonetheless, current definitions and terminology are not ‘conceptually secure’ thereby making any epidemiological work in this field challenging.

### The devil is in the definition

The globally used groups of conditions identified as possibly leading to palliative care of children and young people, are as follows:

1. Life-limiting conditions where cure is possible but can fail (e.g. cancer);

2. Conditions which, though treated intensively over a period of time, inevitably lead to early death (e.g. cystic fibrosis);

3. Progressive conditions where treatment is palliative and often over many years (e.g. muscular dystrophy); and

4. Irreversible but non-progressive conditions giving rise to severe disability and sometimes premature death (e.g. disabilities following brain or spinal cord injury) [1,17].

Estimating the numbers of children from these four groups who actually require palliative care is problematic. The nature of palliative care is that it is defined, not by organ system, but by the needs of an individual child and family. But if children who need specialist palliative care are to be able to have the same access that adults currently enjoy then service developers must engage commissioners with appropriate evidence. That in turn requires better understanding of the numbers of children who need such access, and the proportion of children who will actually use palliative care services at any one time. The challenge facing researchers is, therefore, to provide new ways of producing data that are precise and of practical use, about service needs that are often subjective and individual.

The difficulty in establishing how many children need palliative care is further complicated by the fact that many key terms are not agreed among providers. ‘Palliative care’ encompasses specialist services at and around the end-of-life, but also more generic services valued highly by families at much earlier stages in the disease trajectory. The term ‘life-threatening’, which should properly be reserved for conditions in which premature death is likely but not inevitable, is often used synonymously with the term ‘life-limiting’. Even the term ‘life-limiting’ is not unambiguous, since for some it implies limitation of ability rather than lifespan. Although the globally used Together for Short Lives/Royal College of Paediatrics and Child Health (RCPCH) categories [1,17] provide a measure of consistent categorisation, some lack of clarity remains.

The large variation, and relatively small number of children with palliative care needs, also drives the configuration of services across a wider geographical area. Children’s palliative care and services can be divided into three categories, within which there are a number of important and varied roles [18]:

• Specialist palliative care services – care delivered by specialist providers such as specialist in-patient facilities.

• Core palliative care services – care delivered by people whose primary care focus is palliative care such as community nursing teams.

• Universal palliative care services – care delivered by generalist (non-palliative care specialists) health and social care providers such as General Practitioners and social workers.

Many children requiring palliative care services remain unrecognised by service providers, while research focusing on the individual experiences of children and their families is negligible [19,20]. Although in some areas children’s palliative care provision may be good, in others it is generally unclear who is providing what (if anything), and to whom, thus leading to substantial unmet needs [19-21]. Many children and parents find it difficult or impossible to obtain support tailored to their needs [1] and although leading not-for-profit organisations (our research partners) have focused on improving service delivery, rigorous evidence to inform the commissioning process has been lacking. In addition, commissioners of services are unlikely to be responsive unless methods can be developed to estimate current service costs and costs of commissioning future child-centred palliative care services. The key pieces of the jigsaw that are needed to achieve more rigorous evidence-based commissioning include the need for better understanding of population prevalence of life-limiting conditions versus actual use of palliative care services at any one time, child and parent care preferences and methods for costing services for commissioners, and methods for integrating evidence for use by commissioners.

### Commissioning framework

To promote a more coordinated and joined up approach, the Department of Health England (DH) produced a framework for planning and commissioning children’s palliative care services [22]. The approach incorporates current service provision, secondary analysis of available datasets to estimate numbers of children, ascertaining children’s and parents’ choices and preferred locations of care, and incorporating professional perspectives (see, p.11-12). Whilst the overall framework was helpful in conceptualising the steps in a locality-based needs assessment, it was obvious that additional novel methods of data collection, synthesis and analysis would be needed before the commissioning framework could be used in practice. For example, in the exemplar locality (North Wales), similar to other global contexts, there was no current directory of palliative care services, no database of children with palliative care needs locally, and nor was an advance care planning framework available to ascertain child and parent preferences before the end-of-life stage, or an appropriate costing methodology.

### Conceptual framework for commissioning an integrated palliative care service

**Figure 1:**
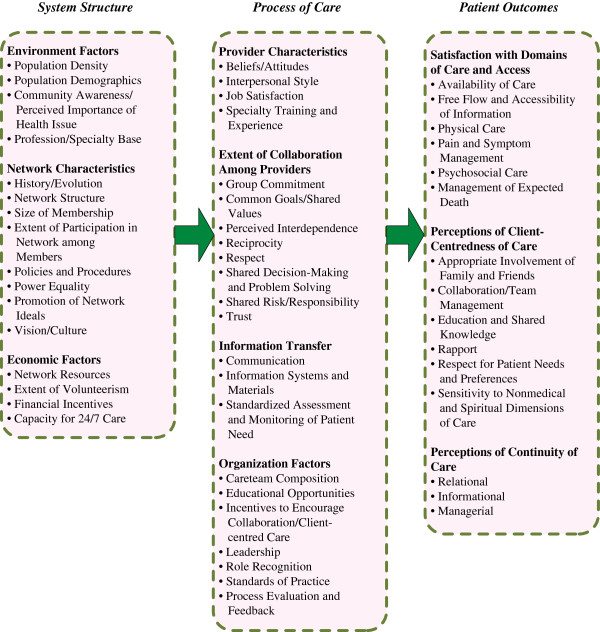
**Conceptual framework for the evaluation of integrated palliative care networks [**[12].

Children’s palliative care needs to be flexible and reactive to the needs of children and parents. It should ideally be integrated and delivered by regional clinical networks, which in the UK are in various stages of development [5]. We used the framework of Bainbridge et al. [12] alongside the DH commissioning guidance [22] to conceptualise the system structure of children’s palliative care within which child, family and client-centred care is a principal construct. The system structure is dependent on environmental factors such as population density and demographics, community awareness and the professional specialty base; network characteristics such as history, size, participation, policies and procedures, power equality, and promotion of vision, ideas and culture; and economic factors such as network resources, volunteerism, financial incentives and capacity for 24/7 care (see Figure 1).

The aim of the study was to use the DH Commissioning guidance [22] as a general framework, and where appropriate integrate existing evidence, and develop or use new methods to generate new evidence to populate a costed commissioning framework. To achieve this we needed to:

• Determine the prevalence of children with life-limiting conditions, and numbers and diagnoses of children and young people up to 19 years dying each year from life-limiting illnesses;

• Identify current services to ascertain the strengths and weaknesses in the managed clinical network and estimate costs of care;

• Develop, implement and evaluate an advance care planning framework called the ‘My Choices’ suite of resources;

• Gain better understanding of children’s and parents’, and bereaved parents’ views, experiences, needs, choices and preferred places of care;

• Ascertain healthcare professionals’ perspectives on services, and

• Identify gaps in current provision and estimate costs of commissioning future child-centred services to fill identified gaps.

In this paper, we report the overarching and interconnected strands of work and show where we needed to integrate existing evidence, develop new methods to populate the commissioning framework with data, integrate new evidence using estimates derived from new methods, and then present the main outcome – the evidence-based costed commissioning framework. More detailed reports of methodological development of the ‘Dictionary’ of life-limiting conditions in childhood [2,3], using the Dictionary to establish population-based prevalence and psychological consequences for children and families [3,23] development and evaluation of the ‘My Choices’ suite of advance care planning resources [24], and qualitative interviews with young people, parents, bereaved parents and healthcare professionals [2] are published in full elsewhere.

## Methods

### Setting

A range of providers of children’s complex health and palliative care within the National Health Service and by not-for-profit organisations providing services to 680,000 people in North Wales of whom approximately 156,600 (23%) are aged 0 – 19 years [25].

### Participants

Children and young people age 0–19 years with complex health and palliative care needs as defined by the Together for Short Lives/RCPCH categories [1,17], parents, and multi-agency palliative care professionals.

### Design

A multi-phase mixed-methods design with the following integrated studies, and using estimates derived from additional novel methodological development, to create an evidence-based and costed commissioning framework, including:

1. Secondary analysis of death certificates to establish numbers and life-limiting diagnosis at death (all-Wales and North Wales) in different age groups and where children and young people who live in North Wales die;

2. Application of three population prevalence estimation methods to determine a) the number of children with life-limiting conditions likely to access hospital services as an in-patient in a given year [3], b) the total population prevalence of children with a life-limiting condition [23], and c) the number of children dying each year [26];

3. Development and evaluation of a child and parent-held future care planning framework (The ‘My Choices’ suite of booklets) to support future care planning and to ascertain children’s and parents’ care choices and preferred locations of palliative care [2];

4. Qualitative interviews, including a task to rank desired service attributes, with young people, parents, bereaved parents and healthcare professionals (with additional questionnaire), to ascertain different stakeholder perspectives on future care planning, service provision and care delivery within a North Wales context [2];

5. Identification and costing of current health and not-for profit organisation children’s palliative care service provision in North Wales;

6. Synthesis of evidence to prioritise service gaps, integrate evidence and develop a costed commissioning framework focusing on targeted identified gaps (children’s community nursing to provide end-of-life care at home, and access to 24 hour support).

### Data collection and analysis

#### Ascertaining life-limiting diagnosis and location at death from death certificates

We undertook an analysis of death certificates to establish diagnosis and location at death in collaboration with Public Health Wales NHS Trust. Many details are recorded on a death certificate including age at death, place of death and underlying cause. Death certificate data for all deaths between 0–19 years in Wales (2002–7) and North Wales (2002–6) (latest cleaned data) were obtained. The North Wales area was defined as the six counties of Anglesey, Gwynedd, Conwy, Denbighshire, Flintshire, and Wrexham.

We have previously developed and validated a ‘Dictionary’ that assigns ICD-10 codes to life-limiting conditions in childhood. The ‘Dictionary’ of life-limiting conditions [2,3] was first compared with age and cause of death on all Wales death certificates (2002–7). For North Wales (2002–6) we looked at age, cause and location of death in North Wales residents, irrespective of place of occurrence, and did not include deaths occurring in North Wales to non-residents.

Data provided by Public Health Wales NHS Trust were organised by age: under 1 month, between one month and 1 year, between 1–6 years, 7–12 years, 13–16 years, and 17–19 years. Underlying cause of death was recorded by ICD-10 Chapters and this information was cross-tabulated with age and place of death. Cause of death was coded into three categories: likely life-limiting medical condition, external causes, and mental and behavioural disorders. External causes included self-harm and suicide and accidental death such as road traffic accidents and other accidents. Deaths were included if medical conditions in ICD-10 Chapters 1 to 14 (excluding Chapter 5 – mental and behavioural disorders) were recorded. Cause of death for inclusion in the study was any medically-related illness. Medically-related illnesses were assumed to be life-limiting in children because any medically-related death had, by definition, limited the child or young person’s life. In analysing the data, we excluded children and young people who died as a result of an external cause, such as an accident, suicide or self-harm, and any child or young person whose death was recorded as resulting from a mental health or behavioural disorder. Neonates were excluded (babies under one month) because they are not typically included in care mapping for palliative care [26].

Death certificate data were tabulated by place of death. Place of death was made available as: hospital, hospice, family home, or elsewhere. Each of the hospital and hospice categories were categorised geographically as: North Wales, Other Wales, and England. It was not possible to gain access to exact underlying cause of death, as identified by ICD-10 code, cross-tabulated with exact place of death as this may have led to identifying information (e.g., there may have been a small number of cases of children dying of a particular medical condition, in a particular hospital) and therefore may have breached the terms and conditions under which Public Health Wales NHS Trust is supplied the data by the Office for National Statistics (ONS).

Any deaths occurring outside of the family home, a hospital or hospice, including those occurring in other types of communal establishments were coded as ‘elsewhere’.

### Modelling

The ‘Dictionary’ of life-limiting conditions has recently been used with ICD-10 codes recorded in Hospital Episode Statistics (HES) to calculate the prevalence of life-limiting conditions in under 19s admitted to hospitals in England in the last year with a life-limiting condition coded in their HES record [3]. Overall prevalence increased from 25/10,000 in 2000/2001 to 32/10,000 in 2009/10. With 30/10,000 age 3 in 2003; 30/10,000 age 5 in 2005 and 22/10,000 age 7 in 2007 (see Table 1). The figures are however likely to underestimate total population prevalence as children with a life-limiting condition may not have been an in-patient within the last year and may not have a life-limiting diagnosis recorded as the reason for admission.

**Table 1:** Analysis of death certificates to establish cause of death and life-limiting condition

**Death certificate analysis age 0 to 19 years 2002-7**	**Overall Picture**						
	**1052 deaths**						
	**569/1052 (54%) deaths from life-limiting conditions**						
	**887 causes of death recorded**						
	**196/887 (22%) causes of death were considered life-limiting.**						
**420/887 children** died from **176** life-limiting conditions	ICD 10 Chapter [27]						
	II - C or D	I- A	IV- E	VI- G	IX- I	X – XVI J or P	XVII- Q
	neoplasms, diseases of the blood and blood-forming organs; certain disorders involving the immune mechanism	infectious and parasitic diseases	endocrine, nutritional and metabolic disease	diseases of the nervous system	diseases of the circulatory system),	diseases of the respiratory system; certain conditions originating in the perinatal period)	congenital malformations, deformations and chromosomal abnormalities
	104/420 (25%)	50/420 (12%)	24/420 (6%)	88/420 (21%)	36/420 (9%)	30/420 (7%)	54/420 (13%)
	Only seven individual life-limiting conditions (4%) caused more than 10 deaths in children						
**169/887 neonatal** deaths from life-limiting conditions	ICD 10 Chapter [27]						
	XVI- P			XVII- Q			
	certain conditions originating in the perinatal period			congenital malformations, deformations and chromosomal abnormalities			
	92/169 (54%)			73/169 (43%)			
	97% deaths were caused by only two life-limiting conditions						

To calculate total population prevalence of life-limiting conditions, in a separate study the ‘Dictionary’ was used to identify the prevalence of life-limiting conditions recorded in the nationally-representative birth cohort at each time point (ages 3, 5, and 7 years) [23]. Total population prevalence of life-limiting conditions was 1.89/10,000 age 3 (2003), 1.45/10,000 age 5 (2005), and 1.44/10,000 age 7 (2007), with an overall prevalence of 1.45/10,000 (Table 1).

Finally, using findings of the report of palliative care services in England [13,26] we used the Lowson et al. model [26], which estimates the number of children and young people who may require palliative care and end-of-life care between 0 and 19 years of age, with the exclusion of neonates (under 1 month old), per capita. The formula to estimate the number of children and young people requiring palliative care is that 15 children and young people per 10,000 have a life-limiting condition that requires access to palliative care service, and that 10% of those will die in a given year.

### Developing and using My Choices booklets to ascertain care preferences

As there was no appropriate existing advance planning framework for children and young people, we developed and evaluated a new and novel set of child and parent-held resources to facilitate thinking and engagement in the planning process, and to determine care preferences and preferred locations of care for children and young people with life-limiting conditions from diagnosis onwards. Development of the My Choices resources has been reported in depth elsewhere [2]. We used the booklets in qualitative interviews with children and parents to inform discussion about their care preferences (see below). The booklets have subsequently been endorsed by Together for Short Lives for routine use in care planning.

### Qualitative interviews with young people, parents, bereaved parents, and interviews and questionnaire with healthcare professionals

To ascertain local service user and stakeholder perspectives on children’s palliative care service delivery and organisation, we interviewed 13 parents and 3 bereaved parents (12 mothers and 5 fathers) who cared for children and young people with complex healthcare and palliative care needs, and 11 children and young people (whose participation varied from active (3) to passive (8) depending on their impairments), and 13 healthcare professionals. With permission, we also took digital photographs of completed My Choices booklets recording care preferences. We also asked parents to rank different service attributes derived from key policy documents in priority order. Interviews were transcribed verbatim and subject to thematic analysis. Twenty-seven healthcare professionals (around half the total local palliative care workforce) also completed researcher designed pre and post questionnaires exploring their attitudes towards different care scenarios and experiences of existing service provision. Questionnaires were analysed with descriptive statistics and open ended questions were subject to content analysis [2]. See Additional files 1 and 2 for a copy of the pre and post study questionnaire.

### Identifying and costing current children’s palliative care services

In a UK context, palliative care for children can be delivered in the following settings by a number of partners from the public, private and not-for-profit sectors, including:

• Hospital setting (general and specialist professionals);

• Hospice (either independent hospices or National Health Service (NHS) hospices);

• Home (through general palliative care providers such as children’s community nurses, or specialist hospice at home services); and

• Local community settings (such as specialist day care).

We consulted local service information and websites, and used our local knowledge and contacts to create a database and map of current services. From a health and not-for-profit organisation perspective, we wanted to cost current children’s palliative care service provision, and to analyse sustainability and implications for future funding. At first attempt, we asked service managers of NHS hospitals, community services, and hospice facilities in North Wales to compare current provision of children’s palliative care across North Wales in terms of posts and unit costs with those of a contemporary review of palliative care services for children and young people in England [13,26]. Using NHS reference costs [28], a schedule was constructed for comparison that described specific elements of Children’s palliative care services across North Wales. Telephone interviews were planned with lead nurses, financial managers and commissioners involved in delivering children’s palliative care in North Wales after they received the schedule. When this approach proved unfeasible – staff did not have this comparative information - we asked heads of services, commissioners and managers to just describe the compliment of staff and other support for children with palliative care needs - so that we might build up the most accurate picture of current palliative care provision in North Wales. With respect to use of hospital services by children with palliative care needs, managers of children’s wards within acute hospital services were approached and asked to supply information about the number of outpatient, inpatient and ward attendances by these children. It was expected that this information could be retrieved from the computerised hospital record system. However, since the admission diagnosis does not always fall into a palliative care category, this approach proved unsuccessful. Instead, individual children’s diagnoses fitting the definition of palliative care were established and the required information were extracted from the hospital database.

NHS-provided services were costed using the Unit Costs of Health and Social Care 2009 [28], the NHS reference costs 2006–2007 (adjusted for inflation) and 2007–2008, and the Agenda for Change pay scale 2008/2009 and 2009/2010. Information about the cost of hospice services was extracted from annual consolidated financial statements.

### Synthesis, priority setting and costing

We synthesised and mapped findings against the conceptual framework of Bainbridge et al. [12], and worked with national and local children’s palliative care leads to set priorities for local commissioning. Using the same costing methodology as described previously, we estimated costs of meeting the priority gaps in current service provision highlighted by parents and young people.

### Ethical considerations

Selected evidence included in this exemplar was drawn from the ‘My Choices’ children’s palliative care study. Approval was granted by the North West Wales Research Ethics Committee, reference number: 08NVNo01/30. Written informed consent to participate in an interview was obtained from parents, young people over 16 years and healthcare professionals. Parent/guardian proxy consent was obtained for young people under 16 years, who also gave their assent to participate. Self-completed questionnaires contained a consent form at the beginning.

## Results

### Diagnoses at death all-Wales

There were 1052 Welsh resident deaths in childhood registered between 2002 and 2007 (see Table 1). Of these, 569/1052 (54%) were from life-limiting conditions as defined by this study. There were 887 causes of death recorded on death certificates of children who lived in Wales in the study period. Of these, 196/887 (22%) causes of death were considered life-limiting. Many life-limiting conditions recorded from death data did not appear in referrals to palliative care services, suggesting under-recognition of and referral to these resources. Outside neonates, 420 children died from 176 different life-limiting conditions. Only seven individual life-limiting conditions (4%) caused more than 10 deaths in children. Among neonates, in contrast, there were 169 neonatal deaths from life-limiting conditions and 97% of deaths were caused by only two life-limiting conditions (see Table 1).

### Annual number of deaths and location of death in North Wales

Around 25 children with life-limiting conditions living in North Wales died per year 2002–2006; 61% of these deaths occurred in North Wales, and 23% of children and young people died in their family home (Tables 2 and 3).

**Table 2:** Mortality statistics between 2002 and 2006 for children and young people living in North Wales, by age and cause of death

**Age of death**		**Medically related deaths**	**Other deaths**	**Total**
1 month to 1 year		38	5	43
1 to 6 years		32	8	40
7 to 12 years		25	5	30
13 to 16 years		15	26	41
17 to 19 years		14	42	56
	Total	124	86	210

**Table 3:** Mortality statistics between 2002 and 2006 for children and young people living in North Wales, by age and place of death

	***Hospital or hospice***	***Family home or other***
*Age at death*		
1 month to 1 year	32	6
1-6 years	26	6
7-12 years	14	11
13-16 years	7	8
17-19 years	7	7
**Total**	**86**	**38**

**Figure 2:**
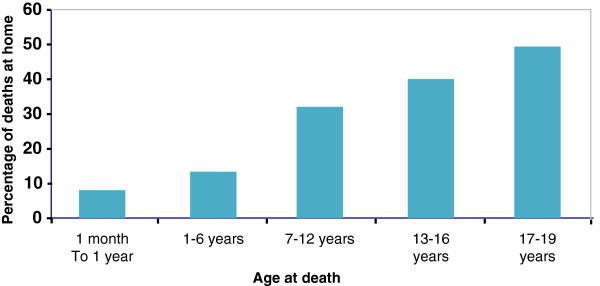
Percentage of deaths of children and young people with a life-limiting condition at home by age.

The place of death for the 124 children and young people who died as a result of medical condition over the period 2002–2006 is summarised in Table 3. Of the 124 children/young people who died, 61% died within the North Wales area; 38% within a North Wales hospital, and 23% within their family home. However, 32% of the children/young people died in hospital or hospice settings in England, which reflects where specialist children’s hospitals are located. Children/young people who died outside of a healthcare facility or their own family home accounted for 8 of the sample. Children were more likely to die at home as they got older, with under 15% of 1–6 year olds dying at home compared to around 50% of 17–19 year olds (Figure 2).

### Modelling using the Dictionary and ICD-10 data

Using the Fraser et al. formula (Table 4) overall estimated prevalence of children with a life-limiting condition experiencing an inpatient stay increased from 405 children in 2000 to 501 in 2009, although this is likely to be an under estimate as not all children will have experienced an in-patient hospital stay in the previous 12 months. Using the Hain et al. formula (Table 4), the total population prevalence in 2009 was 2271 children with a life-limiting condition, although this is likely to be an over estimate. The MCS records only general Chapter headings from the ICD-10, and not the more specific subheadings. In Chapters that encompass conditions that are not life-limiting as well as those that are, the MCS cannot distinguish between them and secondary analysis of MCS data will result in overestimation of prevalence [23].

**Table 4:** Summary table of death certificate number and place of death, and modelling using the Lowson formula, analysis of ICD-10 and MCS with Dictionary of LLCs, and child/parent and professional care preferences

**Epidemiological exemplar for North Wales: children’s palliative care**										
**Data type**	**Comments**	**Year**								
		**2000/1**	**2002/3**	**2003/4**	**2004/5**	**2005/6**	**2006/7**	**2007/8**	**2008/9**	**2009/10**
**Population Estimates North Wales 0 -19 years**^**1**^		**162,100**	**162,100**	**162,100**	**162,100**	**161,300**	**160,000**	**159,200**	**158,000**	**156,600**
**Death certificate analysis 1month – 19 years**	Actual deaths per year		Average deaths **25** per year from a life-limiting condition							
**Death certificate analysis 1month –19 years Place of death**	Actual place of death over 5 years		61% died in North Wales							
			38% North Wales Hospital							
			23% Home							
			32% Hospital/Hospice in England^2^							
**Lowson et al. model 1 month – 19 years**	Estimated deaths per year based on meta-analysis epidemiological evidence/deaths.		Mean population 2002/3- 2007/8							
			161,113 161,113/10,000 (16.09) x 15 = **242** children/10 = **24** deaths per year							
**Prevalence Dictionary/ICD-10-HES** Prevalence of children with LLC in annual contact with hospital as in-patient	***Minimum*** demand by children with LLCs for in-patient services	Overall prevalence 25 per 10,000		30/10,000 Age 3		30/10,000 Age 5		22/10,000 Age 7		Overall prevalence 32 per 10,000
		162,100/10,000 x25 = **405** children								156,600/10,000 x 32 = **501** children
**Prevalence Dictionary/ICD-10-MCS** Prevalence of children with LLC in general population	***Maximum*** number of children who could potentially be affected by a LLC			Overall prevalence of LLC at 3,5 and 7 years 145/10,000						156,600/10,000 x 145 **2270.7** children
				1.89% 189/10,000 Age 3		1.5% 145/10,000 Age 5		1.5% 145/10,000 Age 7		
Parent/child choices interviews									Choice of end-of-life care at home	
Professional perspectives Interviews/questionnaire									Community nursing services too stretched to provide choice	

**Key **^**1**^[29]^*2.*^ Nearest tertiary children’s hospitals are in England. LLC Life-limiting condition.

### Modelling using the Lowson model

Using the Lowson et al. [26] formula, the mean population of children and young people living in North Wales 2002–7 (*n* = 161, 113) was divided by 10,000 and then multiplied by 15 (242, rounded up to the nearest whole number). The projected number of children and young people in North Wales in any given year who may require access to palliative care was estimated to be 242 (Table 4). Lowson et al. [26] estimate that 10% of children with a life-limiting condition will die each year, we therefore divided 242 by 10 (242/10), to calculate an estimate of 24 children and young people and their families may need end-of-life care and bereavement support each year in North Wales - thereby correlating closely with actual death certificate data.

### Young people and parent’s views, preferred service attributes and locations of care, and professional perspectives

Young people and parents experienced fragmented services with insufficient choice or options and described the same gaps as healthcare professionals. Young people’s and parents’ most important service attribute was access to specialist advice and support 24 hours a day. They wanted access to children’s community nursing support for routine complex care and end-of-life care for children at home, and supportive and psychological services for parents and children. Parents usually managed their children at home – even when moderately to severely ill. Parents and young people expressed far stronger preferences for care nearer to home or at home than healthcare professionals. Families valued hospice services for short break care and family support. Professionals had little time for planning ahead, and frequently provided end-of-life care over and above their contracted hours.

Bereaved parents expressed a need for flexible end-of-life service that allowed them to change their preferences at short notice about their child’s location of care between hospital, home and hospice. They also valued continued emotional support after their child had died from professionals who knew them and had cared for them during their child’s life.

### Current multi-agency palliative care service provision and costs

Table 5 shows a summary of children’s palliative care services provided across North Wales. Table 6 summarises current costs, associated with providing children’s palliative care services in North Wales. Nursing costs represent the cost of employment based on the NHS pay scale and do not include travel expenses, which we found difficult to identify and estimate. Costs of hospital attendances exclude specialist procedures and high-cost drugs, which we found impossible to link to children with palliative care needs using the existing computerised hospital record system.

**Table 5:** Summary of children palliative care services provided across 3 hubs in North Wales 2008-2009

**Service**	**North West Wales**	**Conwy and Denbighshire**	**Wrexham and Flintshire**
**Community children's nursing service**	2 WTE band 7	2 WTE band 7	1 WTE band 7
	1 WTE band 6	0.6 WTE band 6	1 WTE band 6
		1 WTE band 4	
**Specialist nurse teams**	0.6 WTE band 7	0.4 band 7	0.8 WTE band 6
Cystic fibrosis service	lead paediatrician	lead paediatrician	1.0 WTE band 7
Cancer	1.0 WTE band 7	1.0 WTE band 7	
		1.0 WTE band 7 (social worker)	
**Hospital services**	Ysbyty Gwynedd	Ysbyty Glan Clwyd	Wrexham Maelor Hospital
admission	214	705	261
outpatient	140	246	71
ward	3		78
**Hospice**		Ty Gobaith	Hope House

**Table 6:** Summary of costs of children palliative care services across 3 hubs in North Wales in 2008-2009

**Service**	**North West Wales**	**Conwy and Denbighshire**	**Wrexham and Flintshire**	**Total**	**Reference**
**Community children's nursing services**	£111355	£120443	£72110	£303908^*^	NHS reference costs 2007-2008
**Specialist nurse teams**					NHS reference costs 2007–2008 and [28]
Cystic fibrosis service	£23547	£15698	£26292	£65537^*^	
Cancer	£39245	£73438	£39245	£151927^*^	
**Hospital services**					NHS reference costs 2006–2007 (adjusted) and 2007-2008
admission	£56774	£187037	£69067	£312877^**^	
outpatient	£26712	£46937	£21176	£94825^**^	
ward	£1567		£40463	£42030^**^	
**Hospice services**			£2487366	**£2487366**	Consolidated financial statements 2008
**Continuing care packages**	£350000	£303079	£1421961	**£2075040**	Information obtained from financial managers
**Total cost**	**£609200**	**£746632**	**£4177680**	**£5533512**	

*cost of employment excluding travel expenses.

**cost exclude specialist procedures and high-cost drugs.

According to our best *maximum* estimate, approximately 2271 children and young people 0–19 years have a life-limiting condition in the total population, and around 501 children are likely to access in-patient treatment each year which equates to the best *minimum* estimate (Table 4). Given the heterogeneous nature of children’s palliative care and increasing life-trajectories, it is logical that there are likely to be more children with life-limiting conditions in the general population than those that access in-patient services in any given year.

Lowson’s model [26] preceeds development of the Dictionary and was based on meta-analysis and death data, and therefore is likely to underestimate population prevalence (242 children), but because of its theoretical foundation, it is likely to provide a more accurate estimate of deaths (24 children requiring end-of-life support in any given year), which was corroborated by actual deaths (25 per year with fluctuations).

The estimated annual total cost of children’s palliative care in North Wales is about £5.5 million, which includes hospital and hospice services, community and specialist nurse teams, and continuing care packages. Approximately half of these costs are paid by the NHS and half by charities, supporting children hospices and community nursing teams. Costs of hospice services and continuing care packages represent approximately 83% of the estimated total cost of children’s palliative care. Continuing care packages for children with palliative care needs (bespoke care packages if child’s needs not met by existing service provision), which include equipment, consumables, nurse and social worker support, were the second largest cost (37%). Hospice services, including end-of-life care, respite care for families and bereavement support, account for 45% of total palliative care costs. According to our estimate, the average care cost per child per year ranged from £22,771 -£11,045-£2,437 (£5,533,512 divided by 243, 501, and 2271 children respectively).

### Priority setting and costing

We synthesised and mapped findings against the conceptual framework [12] and national policy imperatives.

#### System structure: environmental factors, network characteristics and economic factors

We identified a historically fragmented allocation and organisation of resources to this group of children. We found an absence of any robust database/record about expenditure on children’s palliative care in North Wales. At the time that this study was carried out, there were nine primary and secondary care organisations across North Wales, which were subsequently amalgamated into a single organisation, partly explaining why we did not find any single commissioning brief or budget for these children.

All the essential elements of a children’s palliative care network were not yet in place. The most important professional gaps were the lack of a specialist consultant in children’s palliative care based in North Wales, a universal children’s community nursing service, provision of end-of-life care at home, and access to 24/7 support and advice.

In line with a large geographical area with a small population, there were disproportionately more general than specialist palliative care services than seen in larger centres, and those specialist palliative care services such as Diana teams of nurses (children’s community nurses specialising in palliative care) were being absorbed into community nursing teams with a wider remit. Different services commissioned by former organisations also had different remits and eligibility criteria. Services were located in key areas of population with children and families living in rural areas having to travel considerable distances to access services. For services delivered to children at home, the same issues applied with practitioners travelling long distances to deliver care.

#### Process of care: provider characteristics; extent of collaboration among providers, information transfer, organisation factors

Neither parents nor professionals were aware of the totality of local or regional provision, or had a clear idea of the remit, capacity and reach of services. When required, children’s community nursing services tended to provide intensive end-of-life care in children’s homes in substitution to providing routine scheduled children’s community nursing services. There was no capacity in the system to provide both end-of-life care and maintain a routine children’s community nursing service. The children’s hospice in North Wales opened in summer 2004, and provision focused on short break care, family support and end-of-life care.

#### Patient outcomes: satisfaction with domains of care and access, perceptions of client centredness, perceptions of continuity of care

Parent aspirations and care plans matched current policy aspirations for more integrated care nearer to home. Satisfaction with services varied enormously. Lack of choice, lack of consultation, fragmented and insufficient care were common. Children’s community nursing teams and hospice care were highly valued but insufficiently resourced. Home was the preferred choice of location for ongoing care. Unless their child’s condition deteriorated beyond a critical point necessitating hospital admission, parents’ preference was to care for their child at home with support from a responsive 24 hour children’s community nursing service, access to 24 hour advice on symptom and medical management, and out-of-hours prescribing.

Although only 23% of children died at home during 2002–6, parents and young people wanted the choice of end-of-life care at home, although this did not always work out in practice due to lack of resources, organisational issues, or they stayed in hospital as death was imminent and it did not feel appropriate to move location. Evidence from the review of palliative care services in England suggests that with more support at home up to 80% of families would choose for their child to die at home. There was an obvious gap in home-based end-of-life care provision if children and families were to have their preference realised.

### Costing identified gaps in the network

In deciding what aspects of children’s palliative care service provision to prioritise and cost within a commissioning framework, we took into consideration a number of factors including:

• The formation of one new combined primary and secondary health provider from nine previous organisations, which presented opportunities to remove barriers to joint working and to commission services in a strategic way for North Wales;

• Funded places on a post graduate diploma/MSc in palliative care were awarded to local paediatricians so they could dedicate some sessions to specialist children’s palliative care in North Wales – thereby addressing one important gap, and

• The priority for parents, bereaved parents, young people and healthcare professionals was access to specialist advice 24 hours a day, appropriate resources to provide parents with the choice of ongoing care at home with minimal need for hospitalisation, and choice of caring for children at end-of-life at home.

We therefore decided to prioritise children’s community nursing to facilitate an option of end-of-life care for children at home, and access to 24 hour support. In line with the review of palliative care services in Wales [14], we included resources to maintain a registry of children with life-limiting diagnoses and administrative support to a registry, and regular modelling using methods and tools developed for this study to ensure co-ordinated future planning and provision of a regional integrated children’s palliative care service.

### Future additional costs of offering parents a choice of end-of-life care at home

Tables 7 and 8 show the roles, responsibilities and resources required, and our estimates of the additional costs of offering a choice of end-of-life care at home. Based on clinical advice from Hain regarding typical minimum and maximum end-of-life scenarios at home, we estimated a minimum (based on 1 week of end-of-life care for 24 children) additional cost of £14,000 per child per week or a total of £336,000 per year for 24 children to provide 1 week of end-of-life support at home across North Wales. This includes 5.5 WTE children’s community nurses (CCN), trained in palliative care; a 1.0 WTE children’s specialist palliative care nurse to support CCNs; 24/7 telephone nurse consultation provided by children’s community nurses and children’s specialist palliative care nurses; technical support for medical equipment; travel expenses for staff and the provision of psychological support for families. Were end-of-life care to span a longer period (e.g., 4 weeks), then some of the above costs would be substantially higher, e.g. 11.0 WTE community care nurses, increasing the total annual additional costs to £536,500 for North Wales.

**Table 7:** Roles and responsibilities of additional staff to provide choice of end-of-life care at home

**Whole time equivalent (WTE)**	**Role and responsibility**
5.5 WTE	Children’s community nurses, trained in palliative care (assuming 1 week of 24/7 end of life care at home)
1.0 WTE	Children’s specialist palliative care nurse to provide on call support to the children’s community nurses
0.2 WTE	Medical Equipment Technician, responsible for equipment safety and calibration;
0.5 WTE	Clinical Psychologist to provide psychological help for families of children with palliative care needs
0.1 WTE	IT Support Specialist to develop and maintain the database of children who require on-going and end of life palliative care at home
0.5 WTE	Administrator to manage database and organise appointments and consultations for children who on-going and end of life palliative care at home; and to organise inventories of equipment
24/7	Telephone nurse consultation provided by children’s community nurses and paediatric palliative care nurses
	Travel costs (estimated from the literature)

**Table 8:** Summary of proposed additional costs, associated with providing end of life care at home 2010–2011 (assuming 1 week of 24/7, end of life care at home for 24 children)

**Service**	**Pay scale**	**Cost**	**Reference**
**Trained nurses in palliative care**	5.5 WTE Band 6	£194464*	[30]
**Paediatric palliative care nurse**	1.0 WTE Band 7	£42221*	[30]
**Medical Equipment Technician**	0.2 WTE Band 6	£7071*	[30]
**Clinical Psychologist**	0.5 WTE Band 7	£17950	[28]
**IT support**	0.1 WTE Band 7	£4222*	[30]
**Administrative support**	0.5 WTE Band 5	£14435*	[30]
**24/7 telephone nurse consultation**	6.4% of working time (100 hours per annum), nurse WTE Band 7 X 15 nurses	£34500	[28]
**Travel costs**		£20837**	[28]
**Total cost**	**£335700**		

*Midpoint salary plus 20% employment on-costs.

**Adopted from Rapid Response Service travel costs [28] with an average caseload of 364 cases a year.

We acknowledge, however, that estimating length of end-of-life care is a critically under researched and poorly understood area and there is no consistent reported pattern of duration of end-of-life support. Clinical experience suggests that some children only require a week of end-of-life care, but importantly other children can potentially have several episodes of ‘end-of-life’ care, which may last longer than a week for each episode, and some children may require longer than 4 weeks end-of-life care with 24 hour support. We have therefore provided cost estimates for illustrative purposes so that they can be adapted for a local context by multiplying the weekly cost per child for end-of-life care, and adjusted for inflation using annual inflation indices. Additional evidence underpinning the costing process is provided in Additional file 3.

## Discussion

This is the first exemplar that illustrates the challenges of identifying palliative care needs based on additional epidemiological evidence from prevalence estimates using the Dictionary of life-limiting conditions with ICD-10 and HES and MCS data, death certificate data and patient preferences, and costing children’s palliative care service provision using a top down approach as recommended in the Department of Health commissioning guidance [22]. Completing the process would not have been possible without the extensive methodological development to produce new evidence undertaken as part of this study and parallel studies with collaborators using the Dictionary of life-limiting conditions.

We primarily undertook the methodological work because of the difficulty in defining palliative care in children. The adult specialty grew primarily out of cancer management, in which a period of treatment and perhaps remission was followed by relapse and a progression to death that was largely predictable over weeks or months. In contrast, in children the term ‘life-limiting condition’ encompasses non-malignant as well as malignant conditions [1,13,31,32] thus extending the definitions of conditions needing palliative care to include a wide variety of disease trajectories. The question of whether a condition can legitimately be considered life-limiting depends on how these trajectories are seen. At one extreme, palliative care can be seen to be necessary only for children in the last few days or weeks of life. Practically, that approach is unsatisfactory since it means palliative care services are by definition not introduced until death is imminent. This necessarily implies introducing a new medical and nursing team at a time of considerable emotional upheaval for the family.

The globally used Together for Short Lives/RCPCH definitions [1] offer a more satisfactory approach. There are four categories, distinguished from one another by the trajectory of the conditions within each. The categories do not therefore consist of lists of specific disease diagnoses, but a series of narratives that can plausibly describe the trajectory of an individual child with the individual condition. According to this principle, San Filippo’s disease and adrenoleukodystrophy are both categorised in the same group (category III) because they pursue a similar clinical trajectory which, it is argued, imposes a comparable burden on the child and family.

The corollary is that any condition that can be described by one or more of the Together for Short Lives/RCPCH categories is, by definition, life-limiting. Where it is necessary for registration criteria to be precisely defined, the trajectory narratives of the Together for Short Lives/RCPCH categories are too vague. We consider this approach has limited value in providing data for research or for service development unless life-limiting conditions can be mapped against categories as we have done with the Dictionary of life-limiting conditions in childhood. In developing and using the Dictionary, we found that the range of life-limiting conditions referred to, and accepted by, palliative care services in children is much wider than in adults, where the focus has traditionally been on cancer. Nevertheless, the vast majority of life-limiting diagnoses are encompassed in a few hundred diagnostic labels. It was therefore possible in practice to develop a list of them.

Of particular significance is the methodological work to estimate prevalence of children with life-limiting conditions in the UK from death certificate data, HES, and the MCS [3,23]. Secondary analysis of the MCS provides the most precise estimate yet of all children with life-limiting conditions in the population at 3, 5 and 7 years [23]. This methodology can be replicated globally where ICD-10 data is routinely collected in national cohort studies or routine patient data collection. Population-based prevalence estimates are 5–7 times higher than estimates based on children accessing services. A cohort study analysed using a disease classification system identifies every possible case in the population. This is logically greater than the number of children with life-limiting conditions who actually access palliative care services at any given time. Given the definition used in this study, all children identified are likely to need palliative care at some, but not all, of the time in the course of their condition. Previous estimates using the ‘Dictionary’ and ICD-10 data have been based on contacts by children with services, which represents a minimum estimate. Fraser et al. report in 2010 that the prevalence of life-limiting conditions in under 19s was 32 in 10,000 – double the previous estimates derived from death certificate data a decade earlier [13,26]. A doubling of the population over a decade would also require doubling of costs to provide a similar level of care and services to the increased number of children. Our work also confirms that some children who die have not been previously referred to appropriate palliative care services therefore our new estimates are likely to represent maximum population-based prevalence of children with life-limiting conditions and potential to access palliative care at some time in their life-limiting illness. Findings indicate that not all children who require it are able to benefit from palliative care services when appropriate. Further work is required to establish how many and when children would benefit from accessing palliative care services.

Findings also shed new light on the daunting complexity and range of care that children’s palliative care services and children’s community nurses and hospices in particular have to provide. In our analysis of death certificate data, 420 children died from 176 different life-limiting conditions and some of these children may have more than one life-limiting condition. Conditions that are life-limiting in childhood are characterised by marked heterogeneity in their clinical manifestations. Any or all organ systems can be involved, and the spectrum of clinical severity is wide. This variety presents enormous challenges to children’s palliative care teams, who need to be skilled at caring for all children whatever their diagnosis and condition-specific complex needs.

Of particular concern, there were 887 different causes of death among children between 2002 and 2007. Of these, around half were conditions that, in our opinion, could not be described by any of the Together for Short Lives/RCPCH categories. The great majority of these occurred only once in the time period sampled by the death certificate data, and only a handful of diagnoses (7 outside neonates, 5 among neonates) occurred 10 times or more. This confirms our initial impression that a relatively small number of different diagnoses can encompass a large proportion of life-limiting conditions in children.

In the 420 deaths outside the neonatal period with a life-limiting condition, 104 (25%) had cancer. The proportion of children dying from non-malignant life-limiting conditions was higher than in previous studies [19]. The explanation for this difference is probably that our study is the first to be able to identify all children in a population dying from a life-limiting condition. In the past, studies have always relied on reporting by clinicians. The finding of our current survey therefore suggests that there is still under recognition by clinicians of the life-limiting nature of non-malignant conditions. It seems reasonable to assume that this is reflected in an under representation of this group in development of clinical services.

As previously discussed, the nature of the Together for Short Lives/RCPCH categorisation makes it more difficult to decide clearly on life-limiting conditions, since simply by being so young, the chance of surviving to adulthood is smaller than in childhood itself. Although only 5 conditions caused 10 or more deaths per year in the neonatal period, these 5 accounted for 121 out of 169 (72%) of all deaths. The narrow range of life-limiting conditions in the neonatal period causing death suggests that the development of a care pathway that provides a standardised approach to palliative care in this period could be appropriate and effective. Such an approach has been used for some years among adults but, outside the neonatal period, the sheer diversity of conditions limiting life has made it impractical in children. Nevertheless, there is growing interest in developing neonatal palliative care pathways [1] and commissioning new neonatal palliative care outreach hospice services.

On the face of it, it seems remarkable that around half of the conditions from which children died were not deemed by us to be life-limiting. For the purposes of this methodological work, the term ‘life-limiting’ was defined according to whether the disease trajectory could plausibly be described by one or more of the Together for Short Lives/RCPCH categories. It could be argued that if the Together for Short Lives/RCPCH system of categories excludes such a large proportion of causes of death in childhood, it is in fact too restrictive. The categories were defined in order to avoid an inappropriate focus on malignant disease. They were developed, not on the basis of evidence, but of expert opinion. What they purport to have in common is a high probability that the child will not survive into adulthood. This is sometimes expressed by the ‘surprise’ question: ‘Would you be surprised if this child were to survive into adulthood’. This in turn was intended to identify the population of children needing palliative interventions, including financial and psychological support, symptom control and support after death. In other words, what the categories have in common is the need for access to palliative care services, even though at any one time only a proportion of children will have ‘active’ palliative care needs [1].

It could be argued that this is equally true for many other diagnoses that cause death in childhood. The commonest non-palliative cause of death outside neonates is traffic injury. There is no question that this does not fit into any Together for Short Lives/RCPCH category. External causes, accidental and mental health/behavioural diagnoses that are causes of death are different in that early interventions such as accident prevention is needed and as appropriate ongoing treatment from a child and adolescent mental health team. Typically, with these causes of death, there will be no need for specialist symptom control. There might, however, be considerable opportunity for a skilled palliative care team to support the family around difficult decision-making in intensive care, or in help with arranging funerals, or ongoing emotional support after the death has occurred. All of these are legitimate areas of expertise for palliative care.

Respiratory conditions are also prominent among causes of death that are not life-limiting. Again, since these are conditions that do not usually lead to death, it seems reasonable that they are not described by any of the Together for Short Lives/RCPCH categories. At the same time, families of such children will face many similar challenges, including complex decisions, management of physical symptoms, and again help and support around the time of death itself. These examples illustrate the possibility that the globally used Together for Short Lives/RCPCH system of categorisation could be reviewed and expanded. This would necessitate abandoning as the only criterion the ‘surprise’ question. These are children for whom, almost by definition, premature death has come as a surprise. Our analysis of the causes of death of children in Wales between 2002 and 2007 provided further evidence for this.

## Conclusions

It is hard to establish prevalence of children’s palliative care for a number of reasons, but most importantly the diverse nature of clinical providers, and lack of agreement in important definitions. Prevalence data is nevertheless important in order to inform sound economic planning in service development. Having developed the Dictionary of life-limiting conditions for consistently identifying diagnoses that can be considered life-limiting, we were able to use cohort data to establish population prevalence for the first time. Using an existing economic model, we were then able to use that data to develop a costing model for end-of-life care at home. The study provides quantification of the costs of meeting the needs of children dying at home, and therefore also a quantification of the added value provided by palliative care teams in the exemplar locality who provide end-of-life care. Leading not-for-profit organizations are increasingly lobbying for more research in this critically under-researched specialty to develop a stronger evidence-base to inform children’s palliative care globally. Further research is needed to better understand when children are likely to access palliative care services, and the duration and pattern of children’s end-of-life care. Half of children who died did not have conditions that met the globally agreed children's palliative care condition categories, which need revision in light of findings.

## Competing interests

The authors declared that they have no competing interests.

## Authors’ contributions

JN designed the study with RHastings and RHain. VT contributed to analysis of death certificate data and overall project. MD supported development and validation of the Dictionary of life limiting conditions. GRD and CH prepared the death certificate data. VB and LH collected, analysed and interpreted interview and questionnaire data with support from LHS. ML supported development and evaluation of the My Choices booklets and conduct of qualitative fieldwork. JN drafted the manuscript with all authors providing critical review and final approval.

## Authors’ information

JN has experience in child health research, health services research, health economics and evidence synthesis. She specialises in children with complex needs and complex interventions.

RTE has experience in the design, conduct and analysis of economic evaluation of complex interventions, including public health and psycho-social interventions. She has an interest in the economics of children’s services and in particular children living with disability.

RHastings specialises in research with disabled children and adults and their families.

MD is a Consultant Paediatrician with special interest in Paediatric Palliative Medicine.

VB has experience in delivering child health services and is currently a nurse educator.

LH has experience working as a Children’s Community Nurse in Palliative Care and is currently a Paediatric Research Nurse.

RHain is a Honorary Senior Lecturer, Bangor University, Consultant and Lead Clinician Paediatric Palliative Care Children's Hospital, Cardiff, UK.

GRD is Principal Public Health Intelligence Analyst with experience of analysing death certificate data.

CH is Consultant in Public Health with experience of analysing death certificate data.

LHS has experience in educational and child health research, and is currently a research officer.

## Pre-publication history

The pre-publication history for this paper can be accessed here:

http://www.biomedcentral.com/1472-684X/12/18/prepub

## Supplementary Material

Additional file 1 Pre study healthcare professional questionnaire. Click here for file

Additional file 2 Post study healthcare professional questionnaire. Click here for file

Additional file 3 Evidence base for estimates of additional staffing, telephone support and travel costs. Click here for file
