# Supplementary material for: Evidence-based planning and costing palliative care services for children: novel multi-method epidemiological and economic exemplar
Source: BMC Palliat Care. 2013 Apr 25;12:18. doi: 10.1186/1472-684X-12-18 (PMC3651264; doi:10.1186/1472-684X-12-18)
Supplement: Additional file 1 — Pre study healthcare professional questionnaire. [file 1472-684X-12-18-S1.pdf]

My Choices Pre Study Questionnaire

1. Consent Form

Child and family-focussed palliative care: Can advanced planning deliver better commissioning and services?

The 'My Choices' Project

Name of Researcher: Jane Noyes

Please note that we will not be able to include your data in the study if the consent section is not completed

Particpant ID Code: Please note that if this ID label is removed we will be unable to use your data in the study.

.

1. Please enter your full name

Firstname

Surname

2. Please enter today's date:

DD / MM / YYYY

.

Your name, postal address and e-mail address will only be used to contact you when the post study questionnaire is ready for completion.

3. At the end of the study we will ask you to complete another brief questionnaire. Please enter your postal address so that we can forward the questionnaire to you. If you would prefer to receive the second questionnaire by e-mail please enter a current e-mail address.

Address line 1

Address line 2

Address line 3

Post Code

E-mail address

My Choices Pre Study Questionnaire

***4. CONSENT FORM FOR HEALTHCARE PROFESSIONALS.**

Please mark X in the boxes to give your consent.

- [ ] I confirm that I have read and understand the information sheet for the above project and, if appropriate, I have had the opportunity to ask questions.
- [ ] I understand that my participation is voluntary and that I am free to withdraw at any time, without giving any reason.
- [ ] I am happy to fill out a pre and post survey.
- [ ] I agree that any information I give during the study can be used anonymously as quotes or illustrations in the reporting of the study, but I can withdraw the use of any part of the material at any time before the report is published.
- [ ] I understand that any information I give will be anonymised and that my name is only required by the research team to send me the post study questionnaire.
- [ ] I agree to take part in the above study.

2. A Little Bit About You

1. Please circle your choice below:

Are you:

○ **Male** ○ **Female**

***2. Please circle one choice.**

What is your age?

	18-25	26-30	31-35	36-40	41-45	46-50	51-55	56-60	60+
.	○	○	○	○	○	○	○	○	○

***3. Please circle one choice.**

What is your current job role?

○ Teacher
○ Classroom assistant
○ Nurse
○ Doctor
○ Psychologist
○ Health visitor
○ Healthcare assistant
○ Therapist
○ Social worker
○ Portage worker
○ Play specialist
○ Child care worker
○ Healthcare manager
○ Social services manager
○ Other (please state in box below)

Other (please specify)

***4. Please circle one choice.**

In which organisation do you work?

- Health
- Social Services
- Education
- Voluntary Organisation

***5. Please circle one choice.**

Where do you work?

- Community
- Hospital
- School
- Hospice
- Short break
- Other (Please specify in box below)

Other (please specify)

***6. How long have you been working in palliative care?**

Years

Months

My Choices Pre Study Questionnaire

3. Children and Young People's Palliative Care Planning Questionnaire

Palliative care is an essential part of care for many disabled children and young people who have complex health needs. For some children and young people, palliative care is needed only for a short time; for others, it will be the only focus of care from the time a life-threatening or life limiting condition has been diagnosed. Palliative care services can include short-term breaks, counselling, family support services, pain management and symptom control.

Many young people with disabilities and complex health needs transition from around age 16 onwards onto adult services for ongoing support during adulthood. Below are several questions that ask about your responses to future care planning for children receiving palliative care. Please read each question, and circle the button corresponding with the number on the scale that reflects your own views. If your views are described best by the end points of the scale, please circle either number 1 or number 7. If your views are somewhere in between the two end points, please select a position on the scale that reflects where you feel your views should be placed. Please select a response for each of the questions.

1. How confident are you in dealing with the future care planning needs of children and young people receiving palliative care?

	1. Not at all confident	2.	3.	4.	5.	6.	7. Very confident
.							

2. How difficult do you personally find it to discuss the future care planning needs of children and young people receiving palliative care?

	1. Very difficult	2.	3.	4.	5.	6.	7. Not at all difficult
.							

3. To what extent do you feel that the way you deal with the future care planning needs of children and young people receiving palliative care has a positive effect?

	1. Has no positive effect at all	2.	3.	4.	5.	6.	7. Has a very positive effect
.							

4. How satisfied are you with the way in which you deal with the future care planning needs of children and young people receiving palliative care?

	1. Not satisfied at all	2.	3.	4.	5.	6.	7. Very satisfied
.							

5. To what extent do you feel in control of the process of future care planning for children and young people receiving palliative care?

	1. Not in control at all	2	3	4	5	6	7. Very much in control
.							

4. Working with children receiving palliative care and their families in the l...

1. Please consider each of the questionnaire items below and indicate how often each one has applied to you in the past month in your work with children receiving palliative care and their families. Please read each question, and circle the button corresponding to the response that reflects your own views. Please select a response for each of the questions.

	Never	Yes, but infrequently	Yes, a fair amount of the time	Yes, a great deal
I have taken the time to get to know children/young people and their families	○	○	○	○
I have explained future care planning options to parents	○	○	○	○
I have encouraged children/young people to discuss future care planning with their parents	○	○	○	○
I have trusted parents as “experts” on their own child	○	○	○	○
I have explained future care planning options to children/young people	○	○	○	○
I have taken the time to get to know the full range of palliative care services available in my area for children/young people and their families	○	○	○	○
I have answered fully children/young people’s questions about death	○	○	○	○
I have trusted children/young people as knowing what is right for them	○	○	○	○
I have encouraged parents to involve the wider family (e.g., siblings, grandparents) in the discussion of children’s future care planning	○	○	○	○
I have asked parents about their future care planning expectations and wishes	○	○	○	○
I have taken the time to get to know the full range of adult palliative care services available in my area for young people at transition	○	○	○	○
I have encouraged parents to make contact with other families planning for the future care of their child receiving palliative care	○	○	○	○
I have made sure that children/young people receiving care have a chance to say what is important to them in relation to future care planning	○	○	○	○
I have answered fully parents’ questions about their child’s death	○	○	○	○
I have helped parents feel like a partner in their child’s care	○	○	○	○

I have tried to involve the wider family (e.g., siblings, grandparents) in the discussion of children's future care planning	○	○	○	○
I have actively encouraged colleagues to discuss future care planning with children/young people and their families	○	○	○	○
I have encouraged parents to discuss future care planning with their children	○	○	○	○

5. Where should care take place?

1. We are interested in your views on the best place to care for children and young people with palliative care needs. In the table below are some short descriptions of typical scenarios relating to children and young people with palliative care needs. Please read each scenario carefully and for each one indicate your personal view on where children with these needs are best cared for. Please indicate your view by circling the button in the corresponding box. Please select only one response per scenario.

	Children/ young people should always be cared for at home	Children/ young people should mostly be cared for at home	Children/ young people should sometimes be cared for at home	Children/ young people should never be cared for at home
**Short break for parents** Parents frequently care for children with complex healthcare needs on a day to day basis at home. Parents often request a short break away from their child and family home and decisions have to be made about where best to care for the child whilst parents are away.	○	○	○	○
**Mild illness** Children/young people with disabilities and complex health care needs have frequent episodes of mild/moderate illness requiring additional care when vomiting or having diarrhoea, help with eating and drinking and taking extra medicines, and extra monitoring such as having someone sleep in the same room as them at night.	○	○	○	○
**Serious illness** Children/young people with disabilities and complex health care needs have frequent episodes of acute more serious illness requiring such things as artificial feeding (intravenous or naso-gastric), additional more complex medicines (such as intravenous) help with breathing (such as nebulisers and additional physiotherapy), and 24 hour care.	○	○	○	○
**Life threatening illness** Children/young people with disabilities and complex health care needs can experience episodes of life threatening illness requiring 24 hour 1:1 care.	○	○	○	○
**Potential that child/young person may**	○	○	○	○

not recover Children/young people with disabilities and complex health care needs can experience episodes of very serious life threatening illness and doctors consider that they may not recover and may die.

6. Your views and opinions

1. What aspects of palliative care planning with children/young people and their families do you find most challenging?

2. What three things would enable you to improve palliative care planning with children/young people and their families in your organisation?

1.
2.
3.

7. Thank you.

Thank you for completing this questionnaire. Please return it in the pre-paid envelope provided.
