# Supplementary material for: Evidence-based planning and costing palliative care services for children: novel multi-method epidemiological and economic exemplar
Source: BMC Palliat Care. 2013 Apr 25;12:18. doi: 10.1186/1472-684X-12-18 (PMC3651264; doi:10.1186/1472-684X-12-18)
Supplement: Additional file 2 — Post study healthcare professional questionnaire. [file 1472-684X-12-18-S2.pdf]

My Choices Post Study Questionnaire

'My Choices' Questionnaire

During the past 3 months, parents of children and, if appropriate, children in your service have been provided with access to booklets called 'Choices for My Child' for parents and 'My Choices' for children, designed to help parents and their children/adolescents with palliative care needs to consider their options and preferences about future care. Copies of the booklets were also made available to staff within your service. We are interested in your experience of this process and your views on the 'Choices for My Child' and 'My Choices' booklets. Please circle your response to each of the questions.

1. Have you seen uncompleted versions of the 'My Choices' and 'Choices for My Child' booklets and read their contents?

	Yes	No
'Choices for My Child' booklet for parents.	○	○
'My Choices' booklet for children aged 6-11 years.	○	○
'My Choices' booklet for young people aged 12-15 years.	○	○
'My Choices' booklet for young adults over 16 years.	○	○

2. How many times have parents or children/young people shared information with you within their own 'My Choices' or 'Choices for My Child' booklets in the past 3 - 4 months?

- ○ Never
- ○ Once or twice
- ○ Five or more times
- ○ Ten or more times

My Choices Post Study Questionnaire

Feedback on the 'Choices for My Child' and 'My Choices' booklets

1. Please rate the following for the 'Choices for My Child' and 'My Choices' booklets. If you have not read the booklet yourself or have never had information from completed booklets shared with you, please do not answer these questions. Please circle one answer to each question.

	Not helpful at all	A little helpful	Moderately helpful	Very helpful
How useful do you think the 'My Choices' booklets are for children and young people?	◯	◯	◯	◯
How useful do you think the 'Choices for My Child' booklet is for parents and other family members?	◯	◯	◯	◯
How useful do you think the 'My Choices' and 'Choices for My Child' booklets are for staff in palliative care services?	◯	◯	◯	◯
How useful do you think the 'My Choices' and 'Choices for My Child' booklets are overall for helping to plan future care for children and young people with palliative care needs?	◯	◯	◯	◯

Uncompleted versions of the 'My Choices' and 'Choices for My Child' booklets can be viewd at (http://www.bangor.ac.uk/healthcaresciences//research/mychoices.php.en?catid=&subid=7061.

My Choices Post Study Questionnaire

Feedback on the 'Choices for My Child' and 'My Choices' booklets

Please give your thoughts on the 'Choices for My Child' and the 'My Choices' booklet for each age group below.

1. Please give your thoughts on the 'Choices for My Child' booklet for parents. Please circle one answer per question.

	Very poor	Poor	Neither good or poor	Good	Very Good
Presentation of information	○	○	○	○	○
Comprehensiveness of coverage of future care planning issues.	○	○	○	○	○
Ease of Understanding.	○	○	○	○	○

2. Please give your thoughts on the 'My Choices' booklet for children aged 6-10 years. Please circle one answer per question.

	Very poor	Poor	Neither good or poor	Good	Very Good
Presentation of information	○	○	○	○	○
Comprehensiveness of coverage of future care planning issues.	○	○	○	○	○
Ease of Understanding.	○	○	○	○	○

3. Please give your thoughts on the 'My Choices' booklet for children aged 11-15 years. Please circle one answer per question.

	Very poor	Poor	Neither good or poor	Good	Very Good
Presentation of information	○	○	○	○	○
Comprehensiveness of coverage of future care planning issues.	○	○	○	○	○
Ease of Understanding.	○	○	○	○	○

4. Please give your thoughts on the 'My Choices' booklet for young adults aged 16 years and over. Please circle one answer per question.

	Very poor	Poor	Neither good or poor	Good	Very Good
Presentation of information	○	○	○	○	○
Comprehensiveness of coverage of future care planning issues.	○	○	○	○	○
Ease of Understanding.	○	○	○	○	○

My Choices Post Study Questionnaire

My Choices Post Study Questionnaire

Palliative care is an essential part of care for many disabled children and young people who have complex health needs. For some children and young people, palliative care is needed only for a short time; for others, it will be the only focus of care from the time a life-threatening or life limiting condition has been diagnosed. Palliative care services can include short-term breaks, counselling, family support services, pain management and symptom control. Many young people with disabilities and complex health needs transition from around age 16 onwards onto adult services for ongoing support during adulthood. Below are several questions that ask about your responses to future care planning for children receiving palliative care. Please read each question, and circle the option corresponding with the number on the scale that reflects your own views. If your views are described best by the end points of the scale, please circle either number 1 or number 7. If your views are somewhere in between the two end points, please select a position on the scale that reflects where you feel your views should be placed. Please select a response for each of the questions.

1. How confident are you in dealing with the future care planning needs of children and young people receiving palliative care?

	1. Not at all confident	2.	3.	4.	5.	6.	7. Very confident
.	○	○	○	○	○	○	○

2. How difficult do you personally find it to discuss the future care planning needs of children and young people receiving palliative care?

	1. Very difficult	2.	3.	4.	5.	6.	7. Not at all difficult
.	○	○	○	○	○	○	○

3. To what extent do you feel that the way you deal with the future care planning needs of children and young people receiving palliative care has a positive effect?

	1. Has no positive effect at all	2.	3.	4.	5.	6.	7. Has a very positive effect
.	○	○	○	○	○	○	○

4. How satisfied are you with the way in which you deal with the future care planning needs of children and young people receiving palliative care?

	1. Not satisfied at all	2.	3.	4.	5.	6.	7. Very satisfied
.	○	○	○	○	○	○	○

5. To what extent do you feel in control of the process of future care planning for children and young people receiving palliative care?

	1. Not in control at all	2.	3.	4.	5.	6.	7. Very much in control
.	○	○	○	○	○	○	○

My Choices Post Study Questionnaire

My Choices Post Study Questionnaire

1. Please consider each of the questionnaire items below and indicate how often each one has applied to you in the past month in your work with children receiving palliative care and their families. Please read each question, and circle the option corresponding to the response that reflects your own views. Please select a response for each of the questions.

	Never	Yes, but infrequently	Yes, a fair amount of the time	Yes, a great deal
I have taken the time to get to know children/young people and their families	○	○	○	○
I have explained future care planning options to parents	○	○	○	○
I have encouraged children/young people to discuss future care planning with their parents	○	○	○	○
I have trusted parents as "experts" on their own child	○	○	○	○
I have explained future care planning options to children/young people	○	○	○	○
I have taken the time to get to know the full range of palliative care services available in my area for children/young people and their families	○	○	○	○
I have answered fully children/young people's questions about death	○	○	○	○
I have trusted children/young people as knowing what is right for them	○	○	○	○
I have encouraged parents to involve the wider family (e.g., siblings, grandparents) in the discussion of children's future care planning	○	○	○	○
I have asked parents about their future care planning expectations and wishes	○	○	○	○
I have taken the time to get to know the full range of adult palliative care services available in my area for young people at transition	○	○	○	○
I have encouraged parents to make contact with other families planning for the future care of their child receiving palliative care	○	○	○	○
I have made sure that children/young people receiving care have a chance to say what is important to them in relation to future care planning	○	○	○	○
I have answered fully parents' questions about their child's death	○	○	○	○
I have helped parents feel like a partner in their child's care	○	○	○	○
I have tried to involve the wider family (e.g., siblings, grandparents) in the discussion of children's future care planning	○	○	○	○
I have actively encouraged colleagues to discuss future care planning with children/young people and their families	○	○	○	○
I have encouraged parents to discuss future care planning with their children	○	○	○	○

My Choices Post Study Questionnaire

My Choices Post Study Questionnaire

1. We are interested in your views on the best place to care for children and young people with palliative care needs. In the table below are some short descriptions of typical scenarios relating to children and young people with palliative care needs. Please read each scenario carefully and for each one indicate your personal view on where children with these needs are best cared for. Please indicate your view by circling the option in the corresponding box. Please select only one response per scenario.

	Children/young people should always be cared for at home	Children/young people should mostly be cared for at home	Children/young people should sometimes be cared for at home	Children/young people should never be cared for at home
**Short break for parents** Parents frequently care for children with complex healthcare needs on a day to day basis at home. Parents often request a short break away from their child and family home and decisions have to be made about where best to care for the child whilst parents are away.	○	○	○	○
**Mild illness** Children/young people with disabilities and complex health care needs have frequent episodes of mild/moderate illness requiring additional care when vomiting or having diarrhoea, help with eating and drinking and taking extra medicines, and extra monitoring such as having someone sleep in the same room as them at night.	○	○	○	○
**Serious illness** Children/young people with disabilities and complex health care needs have frequent episodes of acute more serious illness requiring such things as artificial feeding (intravenous or naso-gastric), additional more complex medicines (such as intravenous) help with breathing (such as nebulisers and additional physiotherapy), and 24 hour care.	○	○	○	○
**Life threatening illness** Children/young people with disabilities and complex health care needs can experience episodes of life threatening illness requiring 24 hour 1:1 care.	○	○	○	○
**Potential that child/young person may not recover** Children/young people with disabilities and complex health care needs can experience episodes of very serious life threatening illness and doctors consider that they may not recover and may die.	○	○	○	○

My Choices Post Study Questionnaire

Thank you.

Thank you for completing the Post Study Questionnaire. Please return it in the pre-paid envelope provided.
