# Supplementary material for: Evidence-based planning and costing palliative care services for children: novel multi-method epidemiological and economic exemplar
Source: BMC Palliat Care. 2013 Apr 25;12:18. doi: 10.1186/1472-684X-12-18 (PMC3651264; doi:10.1186/1472-684X-12-18)
Supplement: Additional file 3 — Evidence base for estimates of additional staffing, telephone support and travel costs. [file 1472-684X-12-18-S3.docx]

The recommended nurse-to-patient ratios in the UK are based on 3 levels of paediatric high

dependency care [1], where level 3 patient (highest dependency) requires intensive and continuous

supervision, with two or more organ systems needing technological support, including advanced

respiratory support. According to this recommendation, a registered Children's Nurse with an

intensive care qualification will be at the bedside throughout every 24 hour period [1]. Given that we

estimate that in any one year, there will be 24 children across North Wales requiring end of life care,

and that this end of life care at home may range from 1 to 4 weeks in duration (personal

communication Hain), then the above recommended staff to patient ratios suggest the need for

staffing ranging from 5.5 WTE children’s community nurses, where one week of end-of – life care at

home is required, to 11 WTE children’s community nurses, where each child receives 4 weeks of end-of-life care at home, assuming that no more than 2 children require end-of life-care at the same time.

The children’s community nurses will need to be supported by a 1.0 WTE specialist children palliative

care nurse. However, should more than two children require end of life care at the same time,

additional nursing support may be necessary.

We estimated the cost of nurse telephone consultations as 6.4% of working time (100 hours per year)

according to Unit Costs of Health and Social Care [2]. Estimation of travel costs is very difficult, since

it is highly dependent on workload, which varies from case to case. As a closest approximation we

adopted the travel costs of the Rapid Response Service from Royal Victoria Hospital, Folkestone [2].

Note that these travel costs are additional to the existing nurses’ travel costs.

**References**

1. Do Not Attempt Resuscitation (DNAR) policies. NHS. Available at:

http://www.worcestershirehealth.nhs.uk/SWPCT_Library/Policies_and_Procedures/Clinical/D

NARpolicyRevised2006.pdf

2. Curtis L. Unit Costs of Health and Social Care 2009. Available at:

http://www.pssru.ac.uk/uc/uc2009contents.htm
